# Supplementary material for: Quantitative Loop-Mediated Isothermal Amplification Detection of Ustilaginoidea virens Causing Rice False Smut
Source: Int J Mol Sci. 2023 Jun 20;24(12):10388. doi: 10.3390/ijms241210388 (PMC10299090; doi:10.3390/ijms241210388)
Supplement: Supplementary file 1 [file ijms-24-10388-s001.zip › Supplementary Figures S1 and S2.pdf]

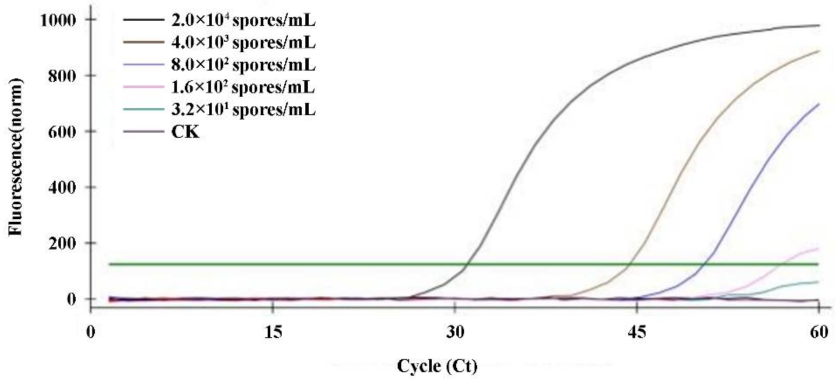

Figure S1. Sensitivity validation of q-PCR detection system. The fluorescence signals in q-PCR detection assays were detected in the samples with DNA template of 2×10^4 spores/mL, 4×10^3 spores/mL, 8×10^2 spores/mL and 1.6×10^2 spores/mL within 60 min, while no signals were detected in sample with DNA template of 32 spores/mL and CK and other spore concentrations. The green line (horizontal) indicates fluorescence threshold. Fluorescence signals above this threshold marked as a successful detection for *U. virens* in q-LAMP assays.

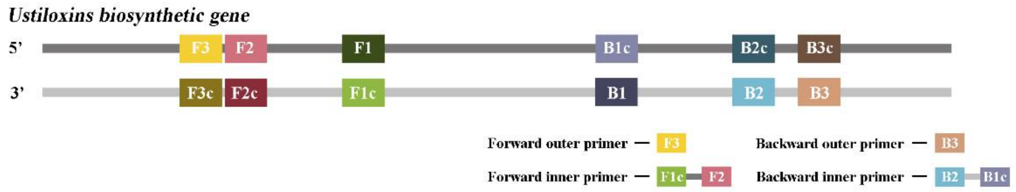

Figure S2. Model for q-LAMP and q-PCR primers design. Primers for q-LAMP assays consist of F3, B3, FIP, and BIP. FIP is a hybrid primer consisting of the F1c and F2 sequences; BIP is a hybrid primer consisting of the B1c and B2 sequences. Primers for q-PCR assays consist of F3 and B3.
