# Supplementary material for: Quantitative Loop-Mediated Isothermal Amplification Detection of Ustilaginoidea virens Causing Rice False Smut
Source: Int J Mol Sci. 2023 Jun 20;24(12):10388. doi: 10.3390/ijms241210388 (PMC10299090; doi:10.3390/ijms241210388)
Supplement: Supplementary file 1 [file ijms-24-10388-s001.zip › Supplementary Table S1.pdf]

Table S1. q-LAMP assay results for standard curve establishment.

Amplification time (x)				The Log_{10} value of spore number (y)
**Repeat 1**	**Repeat 2**	**Repeat 3**	**Mean**	
23.81	27.26	28.73	26.6	6
29.43	31.74	33.15	31.44	5
33.30	35.16	35.58	34.68	4
37.45	38.11	38.20	37.92	3
40.97	40.85	41.66	41.16	2
45.12	42.93	45.15	44.4	1
